# Demographic and life history traits explain patterns in species vulnerability to extinction

**DOI:** 10.1371/journal.pone.0263504

**Published:** 2022-02-23

**Authors:** Haydée Hernández-Yáñez, Su Yeon Kim, Judy P. Che-Castaldo

**Affiliations:** Alexander Center for Applied Population Biology, Conservation & Science Dept., Lincoln Park Zoo, Chicago, Illinois, United States of America; Texas A&M University, UNITED STATES

## Abstract

As ecosystems face disruption of community dynamics and habitat loss, the idea of determining ahead of time which species can become extinct is an important subject in conservation biology. A species’ vulnerability to extinction is dependent upon both intrinsic (life-history strategies, genetics) and extrinsic factors (environment, anthropogenic threats). Studies linking intrinsic traits to extinction risk have shown variable results, and to our knowledge, there has not been a systematic analysis looking at how demographic patterns in stage-specific survival and reproductive rates correlate to extinction risk. We used matrix projection models from the COMPADRE and COMADRE matrix databases and IUCN Red List status as our proxy of extinction risk to investigate if some demographic patterns are more vulnerable to extinction than others. We obtained data on demographic rates, phylogeny, and IUCN status for 159 species of herbaceous plants, trees, mammals, and birds. We calculated 14 demographic metrics related to different aspects of life history and elasticity values and analyzed whether they differ based on IUCN categories using conditional random forest analysis and phylogenetic generalized least square regressions. We mapped all species within the database, both with IUCN assessment and without, and overlaid them with biodiversity hotspots to investigate if there is bias within the assessed species and how many of the non-assessed species could use the demographic information recorded in COMPADRE and COMADRE for future IUCN assessments. We found that herbaceous perennials are more vulnerable when they mature early and have high juvenile survival rates; birds are more vulnerable with high progressive growth and reproduction; mammals are more vulnerable when they have longer generation times. These patterns may be used to assess relative vulnerability across species when lacking abundance or trend data.

## Introduction

Degradation of natural habitats by anthropogenic threats (such as climate change, habitat loss, pollution and poaching) has led to record loss of biodiversity and the acceleration of species extinction rates unprecedented in human history [1]. Extinction is not uncommon and is a process not completely random but influenced by deterministic processes such as biotic interactions, environmental filtering, niche availability, and the species’ biological characteristics [2, 3]. For example, extinction risk can be heightened in animal species with small geographical range size, low local abundance, ecological specialization, large body size, and a high trophic level (e.g. [4–7]).

The ability to predict species vulnerability to extinction has been an essential focus in conservation biology: it would help conservationists identify which species are more prone to extinction and which species could be en route to extinction [8, 9]. Species vulnerability to extinction may depend upon both extrinsic and intrinsic factors. Extrinsic factors are external to the focal species and may be environmental or anthropogenic, such as climate change, habitat loss, and the presence of invasive species [10, 11]. Intrinsic factors are the species’ biological characteristics, such as physiology, behavior and life history. A species’ life history is the pattern and timing of survival, growth, and reproduction over an individual’s life. Differences in life history traits may ultimately determine if populations persist when there is strong environmental variability or extrinsic threats [12]. In plants, differences in the ability to store seeds may strongly influence their survival during bad years [13], which is called the storage effect. Long adult stages, or high adult survival, can potentially maintain long-lived plants through bad years and allow them to withstand demographic stochasticity, for example when there are years with low recruitment rates [14, 15]. Low fecundity and self-fertilization as mating system has also been linked to higher vulnerability to extinction in plants [10, 16, 17]. In mammals, a low reproductive rate, in combination with large body size and isolated populations, have been found to be important contributors to extinction risk [6, 18, 19].

A few theoretical studies have looked at the relationship between extinction risk, stage-specific demographic rates and life history strategies [20, 21]. Life history strategies can be described by patterns in species’ demographic rates: survival, growth, and reproduction [22]. For example, high adult survival, low fecundity, and long generation times are considered a “slow” life history strategy compared to high fecundity and short generations times—a “fast” life history strategy [23, 24]. Jonsson and Ebenman [20] used simulations to study how variability in specific demographic rates affects extinction risk for species with different life history strategies: Certain combinations of life history traits and demographic rates can make a population more prone to extinction than others. They examined patterns using elasticity values, which measure the relative importance of individual demographic rates to overall population growth [25] (for example, a high elasticity value in survival of a specific stage means the population’s growth rate is sensitive to changes in that demographic rate). Jonsson and Ebenman [20] found that iteroparous species (those with multiple reproductive episodes in their lifetime) have high elasticity values for adult survival and are most vulnerable to extinction when adult survival rates fluctuate. For semelparous species (only one reproductive episode in their lifetime), those considered to have a slow life history strategy are most vulnerable to changes in juvenile survival rate. In contrast, those with fast life history traits are most vulnerable to changes in age at maturity. Another study found that extinction risk generally increased with higher fecundity and earlier maturation [21]. For species that mature later, those with higher adult survival rates had higher extinction risk [21]. From these theoretical studies, we predict that species with certain demographic patterns (e.g., the combination of semelparous, few large offspring, and high elasticity values for juvenile survival) would be more vulnerable to extinction than others. To our knowledge, no study has tested these predictions using empirical data on multiple demographic rates for diverse taxa and over a broad geographical range.

In this study, we aim to investigate if certain demographic patterns are more vulnerable to extinction than others using the COMPADRE and COMADRE plant and animal matrix databases [26, 27]. To assess vulnerability to extinction, we used the International Union for Conservation of Nature (IUCN) Red List of threatened species that utilizes the current threats and environmental conditions each species is experiencing. Therefore, our study results and conclusions are based under these current circumstances and we refer to these throughout this manuscript. The COMPADRE and COMADRE databases include extensive demographic information in the form of matrix projection models [25] from the published literature for a multitude of taxa. We gather demographic and phylogenetic information for herbaceous plants, trees, mammals and birds. We test if species vulnerability to extinction, as indicated by their IUCN Red List status, can be explained by patterns in demographic rates. We describe demographic patterns by calculating from the matrix data nine life history metrics [22] and elasticity values measuring the relative importance of adult and juvenile survival to the rate of population growth. We ensured statistical independence across taxa by including phylogenetic information in our analyses.

Additionally, we examined how many species have not been classified by IUCN but have demographic data in COMPADRE and COMADRE that could inform an IUCN status assessment. Because many species have not been assessed due to the lack of data, IUCN assessments could potentially benefit from the data in COMPADRE and COMADRE to inform their analyses. We also hypothesized that the demographic data stored in COMPADRE and COMADRE might be biased towards species in more biodiverse areas; we mapped the species with demographic data and explored whether those with IUCN assessments are more likely to occur in known biodiversity hotspots [28] than species without assessments.

## Methods

We obtained demographic parameters and geographic coordinates of studied populations from the COMPADRE plant matrix database [26] version 6.21.8.0 and COMADRE animal matrix database [27] version 4.21.8.0. We focused on organism types with the most matrix data available: herbaceous perennials, trees, birds, and mammals, and conducted separate analyses for each type. This is because different taxa likely experience different threats, and can also respond to the same threats differently, and therefore the relationship between vulnerability and demographic traits may be dissimilar between taxa even after accounting for phylogeny. We retained population matrices that included fecundity information, can be divided into the three component submatrices (representing survival and growth, sexual reproduction, and clonal reproduction), and had an annual transition time step. We used individual population matrices (those based on data collected in one population and over one time step), but when that is not available for a species we used the mean or pooled matrices, whose values may have been averaged across multiple years or derived from multiple sources as published by the original author.

We gathered the species most current endangered status from the IUCN Red List [29], with five statuses including: Least Concern (LC), Near Threatened (NT), Vulnerable (VU), Endangered (EN), and Critically Endangered (CR). For phylogenetic information, we used the existing phylogeny for herbaceous perennial and tree species in COMPADRE [26] and phylogenies for bird and mammal species from VertLife [30, 31]. For the latter, we inputted a list of the species with both matrix and IUCN data, and sampled 100 trees (the default number) from the “Hacket All Species” distribution for birds and the “node-dated completed trees” distribution for mammals. After combining the population matrix, endangered status, and phylogeny information, our dataset contained 774 matrices across 172 species (Table 1).

**Table 1:** Distribution of species and matrix population model data.

Taxonomic group	Total species	LC species	NT species	VU species	EN species	CR species	Total matrices
Herbaceous perennials	39	20	2	4	6	7	233
Trees	46	26	5	6	5	4	108
Birds	31	20	3	3	2	3	171
Mammals	43	25	3	7	5	3	262

Total number of species included in our analysis across four taxonomic groups and by endangered (IUCN Red List) status.

From each matrix we calculated 14 life history and demographic metrics as possible predictors of extinction risk. The first nine were derived quantities calculated following methods of Salguero-Gómez et al. [22]: degree of iteroparity (whether the species reproduces once or many times in its lifetime), type of survivorship curve, age at maturity, mature life expectancy, net reproductive rate, generation time, mean sexual reproduction, progressive growth, and retrogressive growth (see S1 Table for additional details and definitions). These were calculated from population matrices using the R package *Rage* [32]. The last five metrics (juvenile survival, adult survival, average fecundity, elasticity of juvenile survival, and elasticity of adult survival) were related to stage-specific survival and fecundity as they have been shown to correlate with extinction risk [20, 21]. We standardized calculations of survival and fecundity across the species in our data set by collapsing stages into juveniles (all non-reproductive stages/ages) and adults (all stages/ages that reproduce and older), following [33]. We then calculated juvenile and adult survival by summing the transition probabilities for progression and stasis elements for each stage. For the average fecundity of adults, we first divided the recruitment elements by survival for each parent stage to obtain fecundity (therefore assuming post-reproductive census) before collapsing the reproductive stages [34]. Finally, we also used these collapsed matrices to calculate elasticity values using the *perturb_matrix* function from *Rage* [32]), to obtain values representing the importance of juvenile survival and adult survival to population growth.

To analyze the relationship between the 14 metrics and extinction risk, we used a two-step approach in which we first identified the most important predictors out of the full set of the 14 predictors using a machine learning method (conditional random forest, or crf), and then quantified the effects of the important predictors on IUCN status using regression (phylogenetic generalized least squares regression, or PGLS). The crf is a type of classification and regression tree analysis that can be particularly useful for data exploration as it can handle datasets containing many predictor variables that may be correlated or have complex interactions [35, 36]. This is desirable as we expect our demographic metrics to be correlated. However, its outputs can be difficult to interpret, and the model does not account for phylogenetic relationships, and therefore we apply the second step to quantify the relationship between demographic metrics and extinction vulnerability. In the first step, we fit crfs using the *cforest* function from the R package *party* [35, 36] with 1,000 trees, four predictors tried at each node (mtry = 4, approximating the square-root of the number of total predictors [35]), and default values for all other parameters. We used IUCN status as a continuous response variable (1 to 5, representing LC to CR). Because this method cannot account for random effects, we calculated mean metrics across matrices (e.g., for different populations or time periods) for species with multiple matrices (N = 28 for herbaceous perennials, 11 trees, 13 birds, and 20 mammals), and analyzed the species mean values. To identify the most important predictors of IUCN status, we calculated conditional variable importance values, defined as the difference in model prediction accuracy (i.e., the number of observations classified correctly) before and after permuting a variable averaged across all fitted trees [36]. A larger importance value indicates a greater reduction in model accuracy when the variable is randomly permuted, and therefore higher explanatory power for the original variable. For this, we used the function *varimp* from *party* with the option conditional = TRUE and default values for the other parameters. We defined a predictor variable as important if its variable importance value is at least twice as large as the magnitude of random variation, as indicated by the size of the largest negative value [36].

In the second step, the variables identified as most important in the crf were then further analyzed using phylogenetic generalized least squares (PGLS) regression [37], to estimate their effects on endangered status. For this, we first calculated a consensus tree from the 100 sampled trees for bird and mammal species using the *ls*.*consensus* function from the R package *phytools* [38]; the phylogenetic tree for plant species [26] was already a consensus tree. We fitted the PGLS using the function *gls* from R package *nlme* [39], with the option correlation = corPagel to specify the variance-covariance structure based on the phylogenetic tree for each taxon and estimate Pagel’s lambda, which assesses the strength of the phylogenetic relationship. Predictor variables were standardized (mean = 0 and variance = 1) for the PGLS analysis. Because variable importance values from crf account for interactions between variables, we first included all possible interactions among the predictors in the PGLS, and sequentially removed the interaction effects if they are not significant. We assessed model fit by calculating the percentage of variance explained as 1-(variance of residuals/variance of response). All statistical analyses were performed in R [40].

During our analyses, we noted a large proportion of the species in COMPADRE and COMADRE could not be used in our analyses due to the lack of an IUCN assessment. Therefore, we also summarized how many species have not been assessed by IUCN but have demographic information in those databases that could help inform such an assessment. We also mapped (using ArcGIS Desktop 10.7 [41]) the herbaceous perennials, trees, birds and mammals species using the geographic location available in COMPADRE and COMADRE, and examined if they overlapped with known biodiversity hotspots [28]. Not all species and/or populations have a recorded geolocation (Table 2). For species with multiple populations, to match our previous analyses, we randomly retained only one population per species with geographic information.

**Table 2:** Summary of species in the COMPADRE and COMADRE matrix databases.

Taxon	Total species	Species with IUCN assessment	Percent without IUCN assessment	Species with location	Species with location and IUCN assessment	Percent with location but without IUCN assessment
**Herbaceous perennials**	422	76	82%	311	53	61%
**Trees**	141	111	21%	112	87	17%
**Birds**	85	76	11%	61	55	7%
**Mammals**	128	109	15%	100	89	9%

Species summarized by taxonomic group and whether they have geographic location information and IUCN status assessments.

## Results

**Fig 1:**
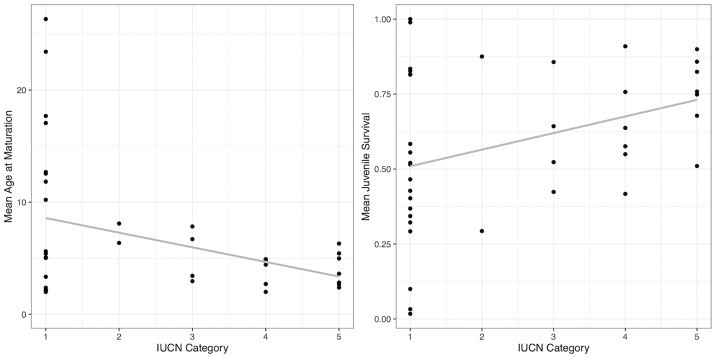
Relationship between endangered status and the most important predictors for herbaceous perennials. The most important predictors of endangered status for 39 herbaceous perennial species are mean age at maturation (left panel) and mean juvenile survival rate (right panel). Trendlines are based on a linear regression of the raw data points and show mean age at maturation decreases with risk, while mean juvenile survival increases with extinction risk. IUCN Red List category (1: Least Concern, 2: Near threatened, 3: Vulnerable, 4: Endangered, 5: Critically Endangered) is used as a proxy of extinction risk.

For herbaceous perennial plants, the crf analysis showed that age at maturity, juvenile survival, and fecundity were important statistical predictors of endangered status (S1 Fig). In the PGLS regression, all possible three-way and two-way interactions between the three variables were not significant (not shown). The model containing only additive effects indicated age at maturity (estimate = -0.81, SE = 0.21, t = -3.76, p<0.001) and juvenile survival (estimate = 0.70, SE = 0.24, t = 2.92, p = 0.006) were significant predictors of endangered status, but not fecundity (estimate = -0.15, SE = 0.24, t = -0.61, p = 0.54), and explained 33.0% percent of the variance. Herbaceous perennial species that begin reproduction earlier and have higher juvenile survival rates tended to be more endangered (Fig 1). We did not find any significant predictors of endangered status in tree species, as the highest variable importance value did not exceed the magnitude of the largest negative value, which represents random variation (S2 Fig).

**Fig 2:**
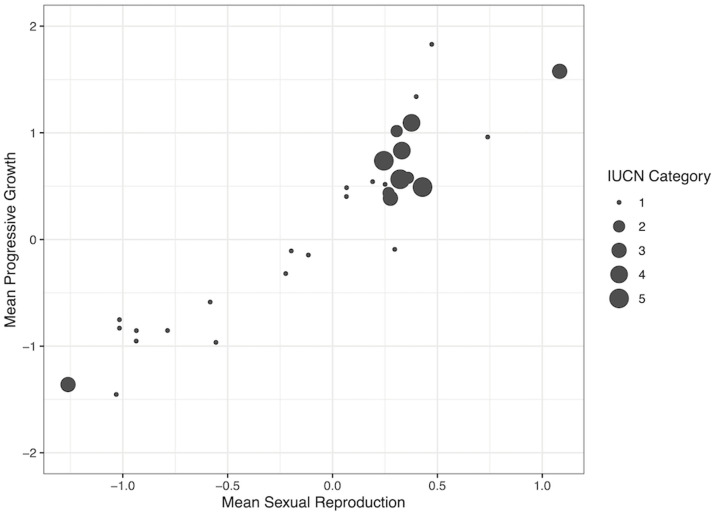
Relationship between endangered status and the most important predictors for birds. The most important predictor of endangered status for 31 bird species is the interaction between reproduction and progressive growth. The size of the circles indicates IUCN Red List category (1: Least Concern, 2: Near threatened, 3: Vulnerable, 4: Endangered, 5: Critically Endangered). Species with high reproduction and growth rates are more likely to have an increased risk (IUCN category).

For bird species, the most important predictors from the crf were reproduction and progressive growth (S3 Fig). The PGLS indicated a significant two-way interaction between these predictors (estimate = 0.67, SE = 0.29, t = 2.35, p = 0.03), but explained only 8.3% percent of the variance. Species with both higher reproduction and greater progressive growth tend to be more endangered (Fig 2). We note that one species, the white tailed eagle (*haliaeetus albicilla*; LC status) is not shown in this figure to improve clarity, due to its very low progressive growth value (-4.1; all species shown in S4 Fig).

**Fig 3:**
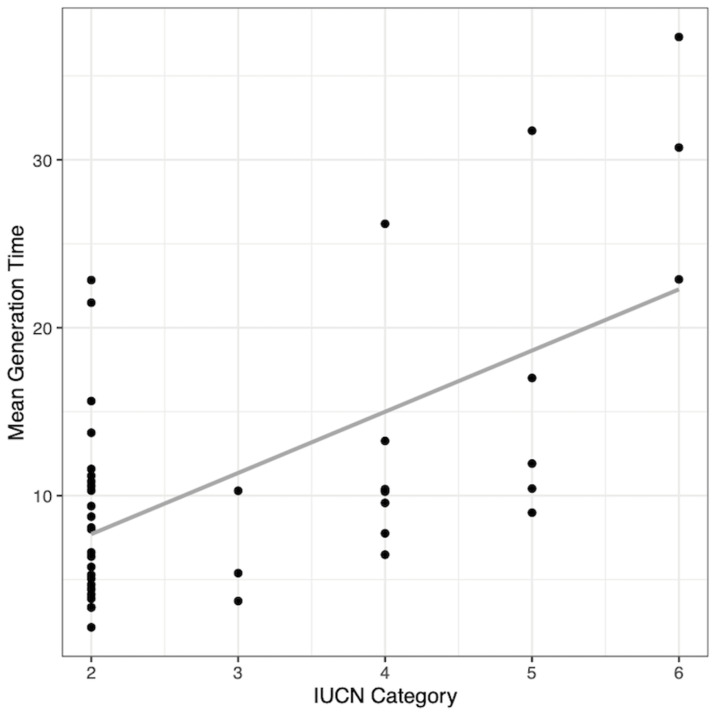
Relationship between endangered status and the most important predictor for mammals. Mean generation time is the most important predictor of endangered status for 43 mammal species and is positively correlated to IUCN Red List categories. See Fig 1 for descriptions of trend lines and IUCN categories.

For mammal species, mature life expectancy, generation time, and adult survival were the most important predictors in the crf (S5 Fig). All possible three-way and two-way interactions between the three variables were not significant in the PGLS regression (not shown). The model containing only additive effects indicated only generation time was a significant statistical predictor (estimate = 0.95, SE = 0.42, t = 2.29, p = 0.03), with longer generation times for species that are more endangered (Fig 3). Mature life expectancy (estimate = -0.09, SE = 0.38, t = -0.23, p = 0.82) and adult survival (estimate = 0.15, SE = 0.22, t = 0.70, p = 0.49) were not significant predictors in the PGLS, which explained 36.7% percent of the variance.

**Fig 4:**
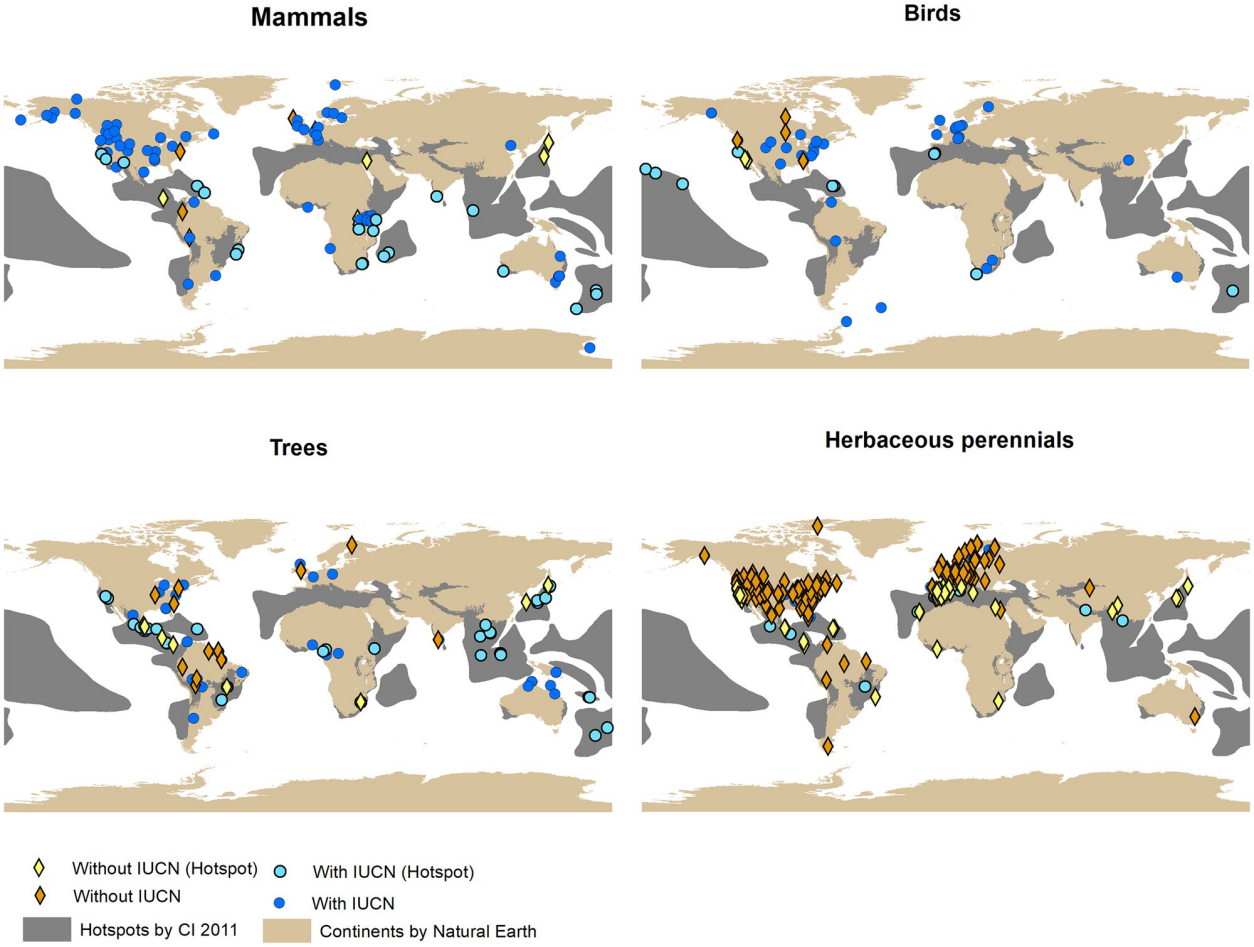
Spatial distribution of species in the COMPADRE and COMADRE matrix databases. Species with and without IUCN status assessment and relative to known biodiversity hotspots. Some species in the databases do not have data on geographic location and therefore were not included in the figure: 28 mammal species (29% without IUCN assessment), 24 bird species (13% without IUCN assessment), 29 tree species (17% without IUCN assessment), and 111 herb species (79% without IUCN assessment).

In total, 404 species with demographic information in COMPADRE and COMADRE did not have an IUCN status assessment (Table 2). The vast majority of these are herbaceous perennial with 346 species. The proportion of species with demographic information that could inform additional IUCN status assessments was smallest for mammals and birds, and intermediate for trees (Table 2; Fig 4). In contrast to our hypothesis, the percentages of species recorded in the COMPADRE and COMADRE databases that are in biodiversity hotspots are low, except for trees (Table 3; Fig 4). The percentages of species without IUCN assessment and inside biodiversity hotspots are also low, except for herbaceous perennials with 70% of the species not assessed (Table 3).

**Table 3:** Percentages of species per taxa with location within biodiversity hotspots.

Taxon	Total species with location	% of species within biodiversity hotspots	% of species within biodiversity hotspots and no IUCN assessment
**Herbaceous perennials**	311	36%	70%
**Trees**	112	53%	15%
**Birds**	61	23%	14%
**Mammals**	100	27%	22%

Species summarized by taxonomic group and the percentages of these species in COMPADRE and COMADRE with location information.

## Discussion

We used the IUCN Red List assessment of threats as a proxy of vulnerability to extinction risk to determine if particular patterns in demographic rates for herbaceous perennial plants, trees, birds, and mammals—calculated from real demographic data—are more prone to extinction than others given the species current environmental conditions. Despite our relatively small sample of species, we found that species with certain demographic patterns are more at risk of extinction than others, and that the important predictors differed between taxonomic groups. For herbaceous perennials, species with earlier maturation (precocious) and higher juvenile survival rates were more vulnerable. Vulnerability of birds was associated with both greater reproduction and progressive growth. Mammals were more prone to extinction with longer generation times. These findings show that, indeed, taxa can differ in the most important variables that explain their vulnerability to extinction, and also that human disturbance (the main extrinsic cause of extinction) does not affect all species equally due to their intrinsic biology [10, 42]. One study that tested the effects of demographic traits such as generation time and age at first reproduction on extinction risk in birds and mammals found that those traits played a small role relative to population size and trends over time [7]. However, accurate estimates of abundance and trends are often not available or costly to obtain [43], and our results show that general demographic patterns may be used to assess relative vulnerability across species in the absence of abundance or trend data. This may be particularly useful for herbaceous perennials, for which a high proportion of species do not have IUCN status assessments (Table 2), and many of these are in biodiversity hotspots (Table 3).

For herbaceous perennials, we found earlier maturation times and higher survival rates in the juvenile stages (i.e., the seedling and vegetative stages prior to reproduction) are correlated with a higher risk of extinction. In simulation studies, earlier maturation generally led to higher extinction risk [21], and particularly for semelparous species with many small offspring [20]. Although perennial plants are not usually semelparous, they produce a large number of offspring (seeds and seedlings) at once compared to birds and mammals. Our results are also consistent with earlier studies that showed shorter life spans correlate with higher extinction risk in plants [17, 44]. Species with shorter life spans, such as biennial plants, have lower adult survival rates [44] and a “fast” life history strategy [22], which can be associated with earlier maturation and higher survival in the pre-reproductive stages. One study that compared common and rare plants did not find a difference in life span, but they did find rare species tended to have lower seed production [16]. Other studies on plants also found measures of reproductive rate to be lower for more vulnerable species [10, 17, 44, 45], so it is interesting that we find survival and maturation rate to be better statistical predictors of vulnerability than fecundity in our analysis (but see discussion of fecundity below).

In the case of birds, higher extinction risk was seen in species with both greater reproduction and progressive growth. These results correspond with predictions from simulation studies, which found that species with early maturation are more vulnerable when they have higher reproduction and intermediate adult survival [21], or when they have many small offspring and are semelparous [20]. Both studies indicate species with higher reproduction and earlier maturation are more vulnerable, but we only found reproduction and not age at maturation as important. It is not necessarily true that species with greater progressive growth (i.e. a higher mean probability of transitioning to a more developed or later stage in the life cycle) will have earlier maturation, in fact our dataset showed these variables were not correlated but trended in the opposite direction (r = 0.32, t_29_ = 1.84, p = 0.08). Therefore, our finding with progressive growth may be a novel result. However, this model explained very little of the variance and thus other, unexplored variables may better explain vulnerability in birds. Overall, there is little agreement in the literature for the relationship between bird demographic traits and extinction vulnerability. Some studies have found that bird species with lower fecundity are more prone to extinction [7, 42, 46], whereas other studies have found no relationship between reproductive rate and bird extinction risk [47–49]. Another study on nearly 8700 bird species showed higher extinction proneness with smaller clutch sizes and earlier developmental mode (precocial rather than altricial), along with other biological and environmental characteristics [50]. Some of the differences could be caused by the many ways in which reproductive rates could be measured. In our analysis, reproduction (the mean per-capita number of recruits across life cycle stages per year) was found to be important but not fecundity (average number of offspring produced by an adult per year). This could be because these variables are correlated, and reproduction had a larger independent effect than fecundity based on our dataset. It could also be due to our estimates of fecundity, which assumed that the original authors of the matrices all used a post-reproductive census and correctly calculated the corresponding recruitment elements. Unfortunately many errors can occur in the calculation of these elements [51], and we also lack information on which type of census (pre- or post-reproductive) was conducted for each matrix. These data are now starting to be collected [52] but our results related to fecundity (for all taxa) are certainly affected by these assumptions, and we suggest revisiting these analyses when that information becomes available.

We found that extinction risk in mammals increased with longer generation times, which is consistent with earlier findings that slow life histories are associated with a high extinction risk in mammals broadly [53] and in carnivores and primates [5]. We did not find relationships based on our reproductive traits of net reproductive rate, mean reproduction, and degree of iteroparity. This is in contrast to the majority of previous studies on mammal extinction risk, which found higher vulnerability for species with lower reproductive rates [5, 7, 19, 54]. However, some studies also showed the opposite [54] or no relationship [18, 19] between reproductive traits and extinction risk, so the relationships may again depend on the specific measure of reproductive rate, as well as the set of species examined. For both herbaceous perennials and mammals, there were traits identified as important in the crf but not significant in the PGLS. This suggests that the effects of those traits were no longer significant when accounting for phylogenetic relatedness between species. For example, in the case of mammals, there may be species that have high adult survival and are more threatened, but they are closely related so that across the phylogenetic tree there is not a strong relationship between adult survival and endangered status.

We did not find statistical predictors of extinction risk in trees, even though the distribution of the different endangered statuses were similar in trees compared to that for other taxa (i.e., primarily Least Concern; Table 1). Tree species may all have similar life histories—long generation times, high adult survival—and there may not be enough variation in life histories to detect differences between endangered statuses. It is also possible that for trees, extrinsic factors have more of an influence on extinction than their intrinsic biology. After all, deforestation for growing crops and urbanization do not discriminate among tree species. Similarly, some of our results, such as longer generation time in mammals and higher reproduction in birds, are opposite in direction to expectations from theoretical studies on species’ ability to withstand environmental fluctuations [22, 55]. This could be due to the species facing extrinsic threats that affect particularly sensitive vital rates. For example, species with longer lives and generation times should be buffered from environmental changes, but they could be vulnerable if the threats they face affected survival (such as overexploitation), which is the vital they are most sensitive to. We also did not find patterns based on elasticities even though species with high elasticity values for juvenile and adult survival may be more prone to extinction [20]. This could be because the actual rates of juvenile and adult survival observed in real populations are less extreme than the values used in the simulated study. Therefore, the relative impact of changing those demographic rates on population growth did not differ between species with different endangered statuses. Finally, we recognize there are alternative methods of analysis that could be used to assess relationships between demographic traits and IUCN status. We explored one such method, a phylogenetically-informed principle components analysis (PCA: S1 File). In general, the results agreed with our main analysis, identifying juvenile survival as an important predictor for herbaceous perennials, no important predictor for trees, reproduction and growth as important predictors for birds, and generation time as an important predictor for mammals; indicating these results are robust. For all taxa except trees, the PCA method also identified additional predictors, and we include these results as Supporting Information (S1 File).

Overall, however, we were able to find statistical predictors that could explain extinction vulnerability under current conditions despite a relatively small number of species (which is still similar in scale to previous analyses, e.g., [7, 18]). Our sample size was due to few species having all three types of data needed for our analysis: demographic information in the form of matrix population models, IUCN assessments for endangered status, and phylogenetic information. We therefore consider it important to use the demographic data available to carry out IUCN assessments for the remaining species. The COMPADRE and COMADRE databases contain detailed demographic information for many species currently without IUCN assessments, particularly herbaceous perennial plants and trees (Table 2). In contrast to our hypothesis, species with location data in COMPADRE and COMADRE were biased towards areas that are not biodiversity hotspots (Fig 4). This is consistent with a recent finding that demographic studies tend to take place in less biodiverse areas [56]. An exception appears to be that demographic studies for trees are evenly distributed among hotspots and non-hotspots, at least those with geolocations recorded. However, low percentages of species without IUCN assessments were found within biodiversity hotspots (Table 3). This is consistent with the reason of why hotspots are important according to conservation international: they are areas that are highly threatened (biodiversityhotspots.org) and have the most studied species, at least this is the case within COMPADRE and COMADRE. The one exception are herbaceous perennials plants, with most of the species not assessed by IUCN being in hotspots. Out of the herbaceous perennial plants in COMPADRE, 258 species have a location but are without IUCN assessment, and out of those 84% are within hotspots. We can conclude that all species missing IUCN assessment due to lack of data on the species could benefit from the demographic information in COMPADRE and COMADRE to inform future IUCN assessments and extinction statuses, but most specifically herbaceous perennial plants.

In addition to applying the existing demographic data in IUCN assessments, a subsequent study could use the demographic relationships we found here to provide a possible endangered status for species in COMPADRE and COMADRE but without IUCN assessments, and identify species that are potentially at risk of extinction. Future analyses could also use the geolocations to compile information on extrinsic factors such as human population density and level of urban development to examine the relative importance of extrinsic versus intrinsic factors on vulnerability to extinction.

## Supporting information

S1 Fig Variable importance values from the conditional random forest analysis for herbaceous perennials. These were used to predict IUCN endangered status (continuous response from 1 to 5, representing LC to CR) based on 14 life history and demographic traits for 36 herbaceous perennial species. The variable importance values are calculated as the difference in model prediction accuracy before and after permuting a variable, averaged across all fitted trees [34]; the variables with the highest importance values have the largest independent effects on endangered status. We defined a predictor variable as important if its variable importance value is at least twice as large as the magnitude of random variation, as indicated by the size of the largest negative value [34]. See S1 File for descriptions of trait abbreviations.
(TIF)

S2 Fig Variable importance values from the conditional random forest analysis for trees. These were used to predict IUCN endangered status (continuous response from 1 to 5, representing LC to CR) based on 14 life history and demographic traits for 48 tree species. See S1 Fig for a definition of variable importance. In this case, no predictors are considered significant as the negative importance values have the largest magnitudes.
(TIF)

S3 Fig Variable importance values from the conditional random forest analysis for birds. These were used to predict IUCN endangered status (continuous response from 1 to 5, representing LC to CR) based on 14 life history and demographic traits for 43 bird species. See S1 Fig for a definition of variable importance.
(TIF)

S4 Fig Relationship between endangered status and the most important predictors for birds. This figure is the same as Fig 2, but includes one additional species (white tailed eagle; *haliaeetus albicilla*) so that all 31 species of birds included in our PGLS analysis.
(TIF)

S5 Fig Variable importance values from the conditional random forest analysis mammals. These were used to predict IUCN endangered status (continuous response from 1 to 5, representing LC to CR) based on 14 life history and demographic traits for 118 mammal species. See S1 Fig for a definition of variable importance.
(TIF)

S1 Table (DOCX)

S1 File (DOCX)
