# Supplementary figures and images for: Demographic and life history traits explain patterns in species vulnerability to extinction
Source: PLoS One. 2022 Feb 23;17(2):e0263504. doi: 10.1371/journal.pone.0263504 (PMC8865652; doi:10.1371/journal.pone.0263504)

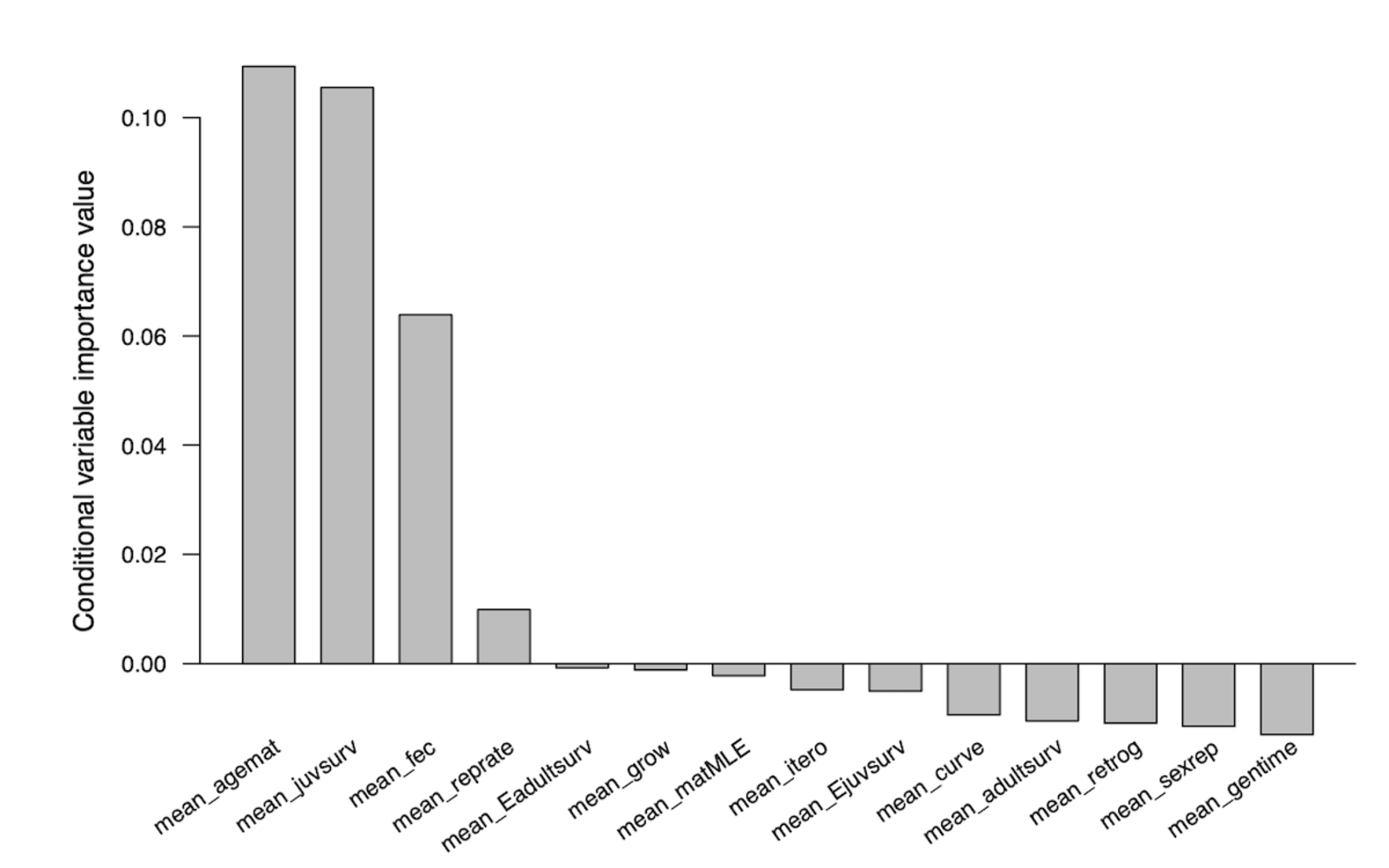

Supplement: S1 Fig — These were used to predict IUCN endangered status (continuous response from 1 to 5, representing LC to CR) based on 14 life history and demographic traits for 36 herbaceous perennial species. The variable importance values are calculated as the difference in model prediction accuracy before and after permuting a variable, averaged across all fitted trees [34]; the variables with the highest importance values have the largest independent effects on endangered status. We defined a predictor variable as important if its variable importance value is at least twice as large as the magnitude of random variation, as indicated by the size of the largest negative value [34]. See S1 File for descriptions of trait abbreviations. (TIF) [file pone.0263504.s001.tif]

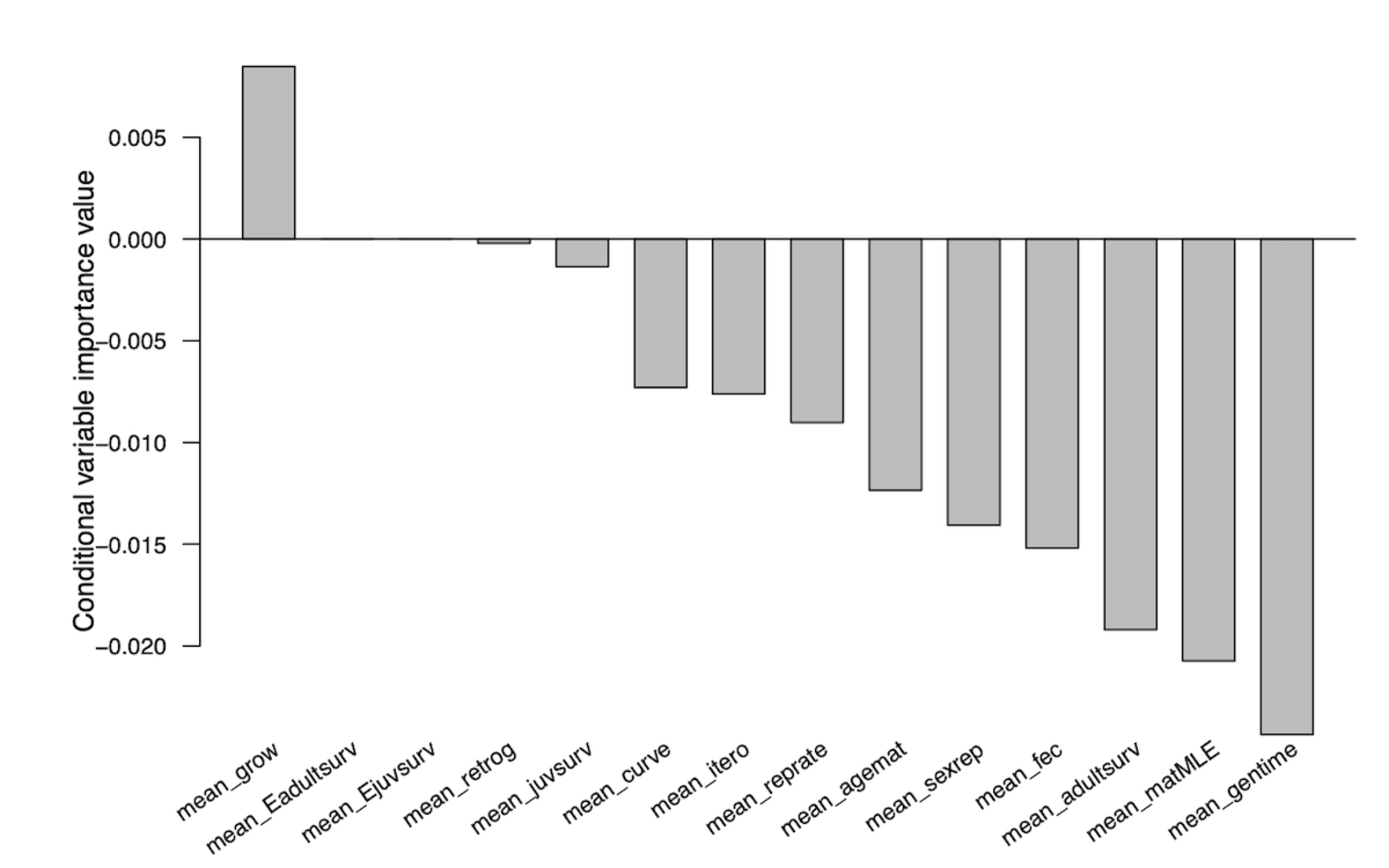

Supplement: S2 Fig — These were used to predict IUCN endangered status (continuous response from 1 to 5, representing LC to CR) based on 14 life history and demographic traits for 48 tree species. See S1 Fig for a definition of variable importance. In this case, no predictors are considered significant as the negative importance values have the largest magnitudes. (TIF) [file pone.0263504.s002.tif]

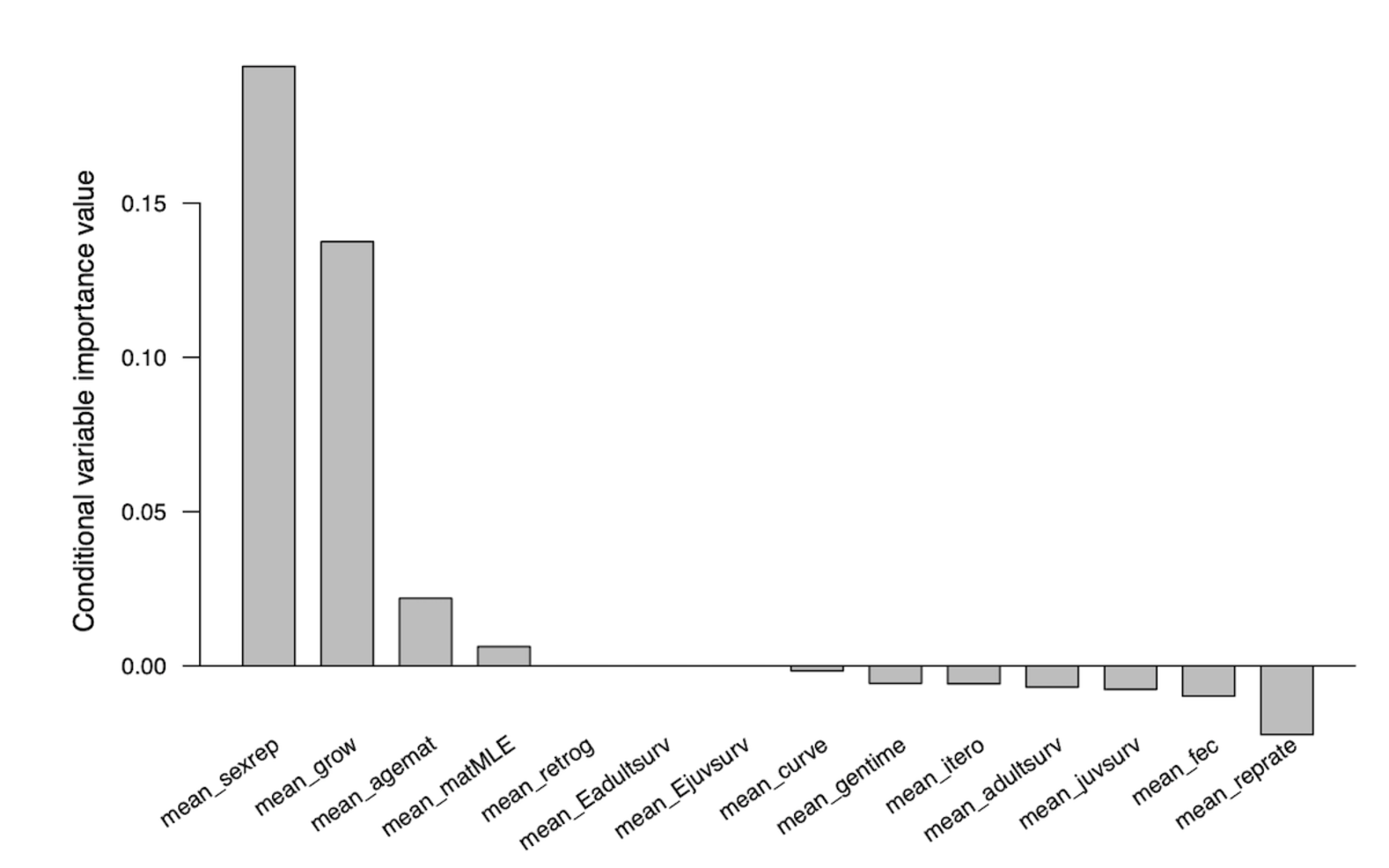

Supplement: S3 Fig — These were used to predict IUCN endangered status (continuous response from 1 to 5, representing LC to CR) based on 14 life history and demographic traits for 43 bird species. See S1 Fig for a definition of variable importance. (TIF) [file pone.0263504.s003.tif]

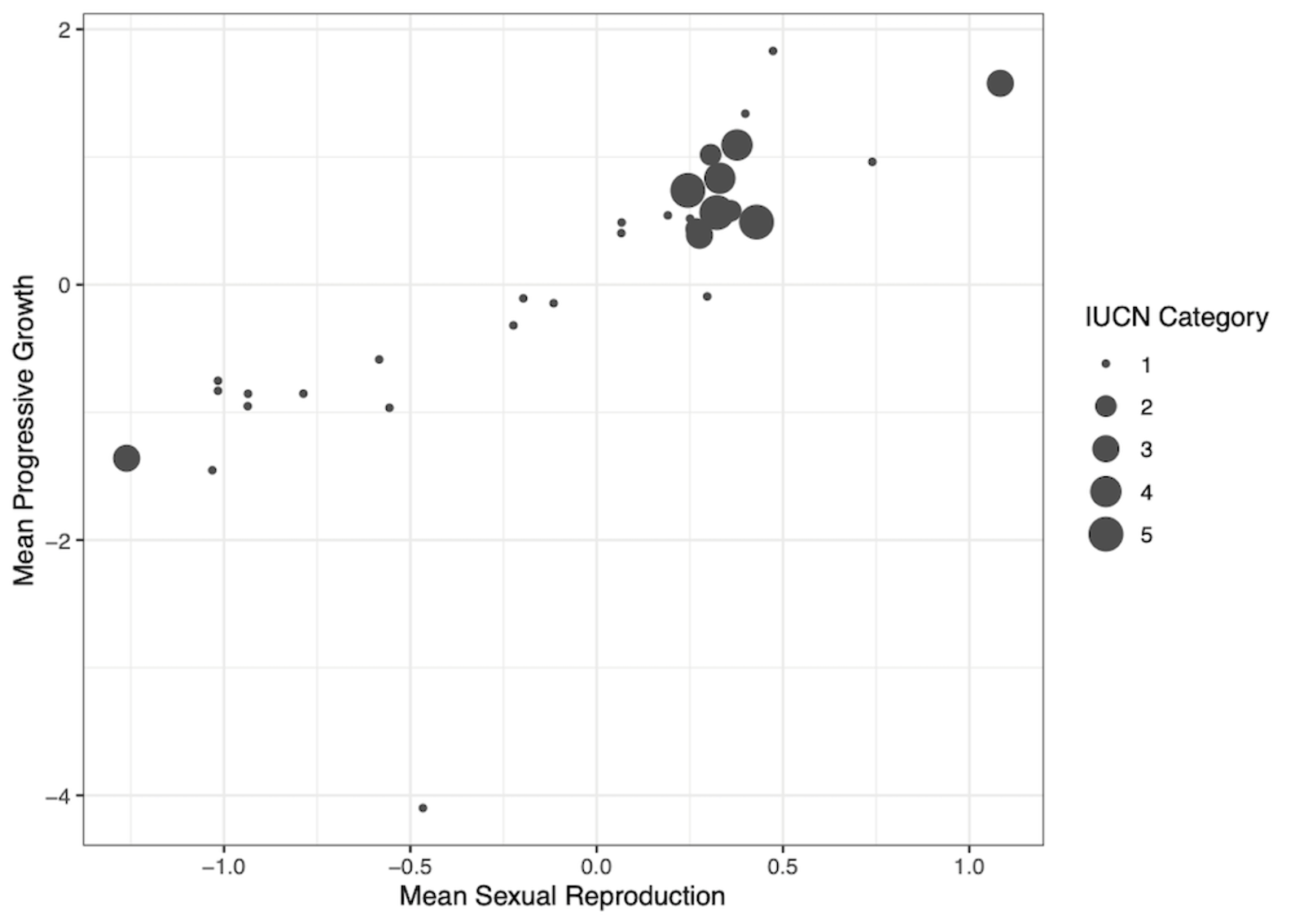

Supplement: S4 Fig — This figure is the same as Fig 2, but includes one additional species (white tailed eagle; haliaeetus albicilla) so that all 31 species of birds included in our PGLS analysis. (TIF) [file pone.0263504.s004.tif]

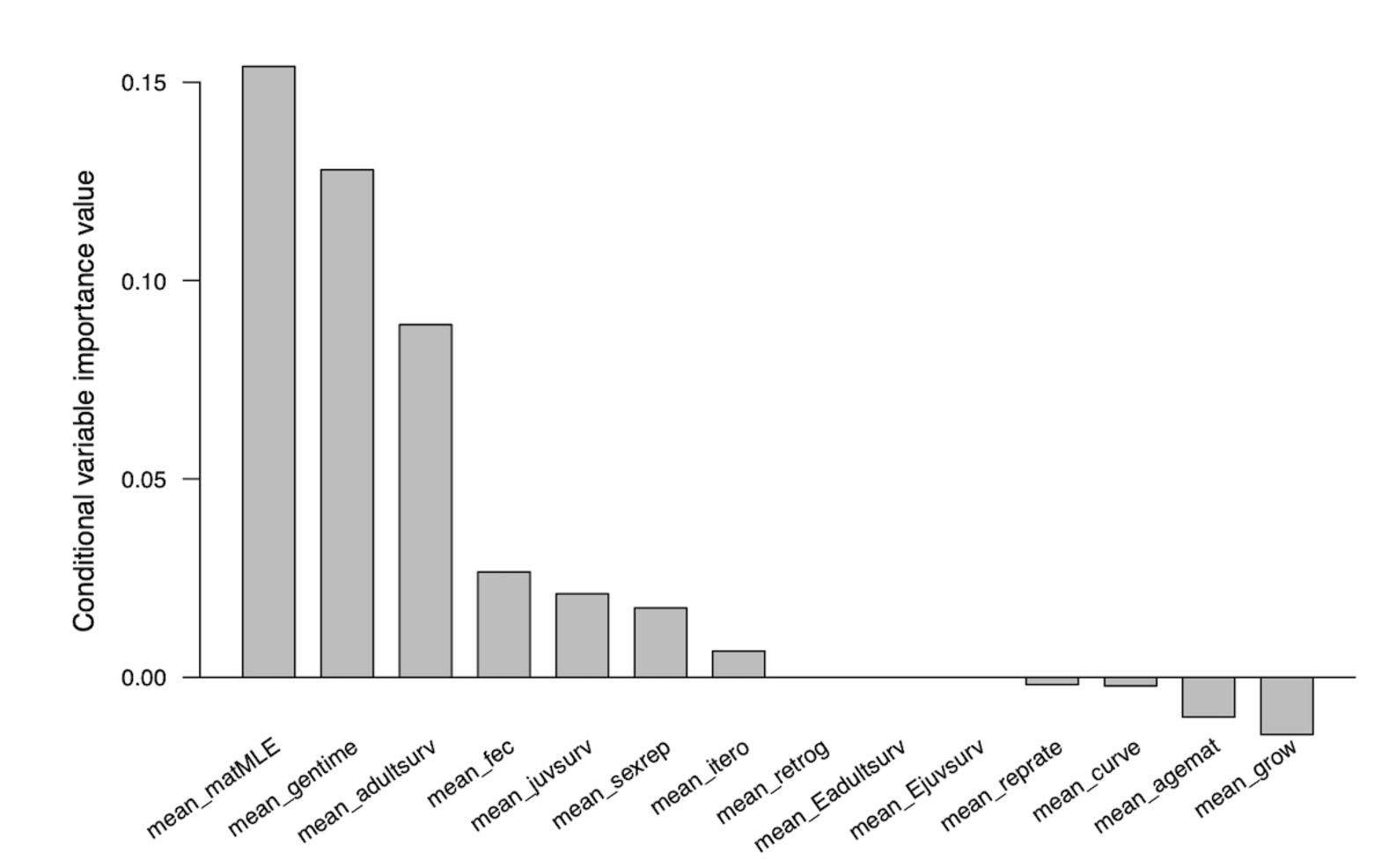

Supplement: S5 Fig — These were used to predict IUCN endangered status (continuous response from 1 to 5, representing LC to CR) based on 14 life history and demographic traits for 118 mammal species. See S1 Fig for a definition of variable importance. (TIF) [file pone.0263504.s005.tif]
