# Supplementary material for: Demographic and life history traits explain patterns in species vulnerability to extinction
Source: PLoS One. 2022 Feb 23;17(2):e0263504. doi: 10.1371/journal.pone.0263504 (PMC8865652; doi:10.1371/journal.pone.0263504)
Supplement: S1 Table — (DOCX) [file pone.0263504.s006.docx]

Supporting Information for Demographic and life history traits explain patterns in species vulnerability to extinction

Haydée Hernández-Yáñez, Su Yeon Kim, Judy P. Che-Castaldo

Supporting Information S1 Table

| **Traits** | **Definition** |
| --- | --- |
| Degree of iteroparity | Spread of reproduction throughout the lifespan of the individual as quantified by Demetrius’ entropy (S). High and low S values correspond to iteroparous and semelparous populations, respectively |
| Type of survivorship curve | Age-specific survivorship curve quantified by Keyfitz’s entropy (H). H>1, =1, <1 correspond to survivorship curve types I. II, and III, respectively |
| Age at maturity | Number of years that it takes an average individual in the population to become sexually reproductive |
| Mature life expectancy | Number of years from the mean age at sexual maturity to the mean life expectancy of an individual in the population |
| Net reproductive rate | Mean number of recruits produced during the mean life expectancy of an individual in the population |
| Generation time | Number of years needed for the individuals of a population to be fully replaced by new individuals |
| Mean sexual reproduction | Mean per-capita number of sexual recruits across stages in the life cycle of the species, weighted by the stable stage distribution |
| Progressive growth | Mean probability of transitioning to a larger/more developed stage in the life cycle of the species, weighted by the stable stage distribution |
| Retrogressive growth | Mean probability of transitioning to a smaller/less developed stage in the life cycle of the species weighted by the stable stage distribution |

Detailed definitions for the nine life history metrics calculated from the matrices in our dataset. Table and definitions taken from Salguero-Gómez et al. [1].

Literature cited:

1. Salguero-Gómez R, Jones OR, Jongejans E, Blomberg SP, Hodgson DJ, Mbeau-Ache C, et al. Fast-slow continuum and reproductive strategies structure plant life-history variation worldwide. Proc Natl Acad Sci U S A. 2016;113(1):230–5
