# Supplementary material for: Demographic and life history traits explain patterns in species vulnerability to extinction
Source: PLoS One. 2022 Feb 23;17(2):e0263504. doi: 10.1371/journal.pone.0263504 (PMC8865652; doi:10.1371/journal.pone.0263504)
Supplement: S1 File — (DOCX) [file pone.0263504.s007.docx]

Supporting Information for Demographic and Life History Traits Explain Patterns in Species Vulnerability to Extinction

Haydée Hernández-Yáñez, Su Yeon Kim, Judy P. Che-Castaldo

Supporting Information S1 File

Alternative data analysis using phylogenetic PCA

*Methods and Summary*

We explored an alternative method of data analysis to examine the relationship between species IUCN status and demographic traits, using the species-mean dataset and phylogenetic information from the main analysis. We implemented a phylogenetically-informed principle components analysis (PCA) using the function *phyl.pca* from the R package *phytools* [1]. For this, we applied the option to calculate the lambda parameter to estimate the strength of the phylogenetic relationship (method = “lambda”), and to use the correlation matrix (mode = “corr”) as our predictor variables have differing scales. We then used the resulting PCs in the conditional random forest (crf) analysis to determine the PC with the largest effects on IUCN status (a continuous response variable ranging from 1 to 5, representing LC to CR). We fit crfs using the *cforest* function from the R package *party* [2,3] with 1,000 trees, four predictors tried at each node (mtry = 4), and default values for all other parameters. We plotted the partial dependence of the most important predictors to visualize its effects on IUCN status.

For trees, birds, and mammals, the PCA could not run with the full dataset, likely because there was very little variation in the two elasticity parameters: the majority of species in these taxa had elasticity of 1 for adult survival and elasticity of 0 for juvenile survival. Therefore, the analyses for these taxa were run without the elasticity of juvenile survival variable, resulting in 13 total predictors.

Unlike previous studies using PCA to analyze population matrix data [4-6], we did not find clear primary axes (PCs 1 and 2) representing the pace of life and reproductive strategies. This is likely because our species set is smaller and may not represent the full range of life histories well. Overall, however, the results were in agreement with our main analysis for all four taxa. Specifically, both analyses identified juvenile survival as an important predictor for herbaceous perennials, no important predictor for trees, sexual reproduction and growth as important predictors for birds, and generation time as an important predictor for mammals, indicating these results are robust. For all taxa except trees, additional predictors were also identified in the PCA, with these differences being the largest for herbaceous perennials. For those species, generation time, mature life expectancy, and net reproductive rate were found to be important in the PCA whereas our initial analysis identified age at maturation as important. These metrics are certainly related, and we cannot be sure which analysis might produce the more generalizable results.

*Detailed results*

I. Herbaceous perennials

The most important predictors from the crf were PCs 12 and 5. Based on loadings for PC12 and the partial dependence plot, there is a negative relationship so that higher values for survival curve (more similar to Type I survival curve), higher generation time, and lower mature life expectancy were associated with higher-risk IUCN statuses. Based on loadings for PC5 and the partial dependence plot, there is a negative relationship so that higher juvenile survival and lower net reproductive rate were associated with higher-risk IUCN statuses.

| Importance |  |  |  |  |  |  |  |  |  |  |  |  |  |  |
| --- | --- | --- | --- | --- | --- | --- | --- | --- | --- | --- | --- | --- | --- | --- |
|  | PC1 | PC2 | PC3 | PC4 | PC5 | PC6 | PC7 | PC8 | PC9 | PC10 | PC11 | PC12 | PC13 | PC14 |
| Standard deviation | 1.90 | 1.64 | 1.48 | 1.22 | 1.05 | 0.90 | 0.82 | 0.72 | 0.58 | 0.49 | 0.38 | 0.34 | 0.21 | 0.13 |
| Proportion of Variance | 0.26 | 0.19 | 0.16 | 0.11 | 0.08 | 0.06 | 0.05 | 0.04 | 0.02 | 0.02 | 0.01 | 0.01 | 0.00 | 0.00 |
| Cumulative Proportion | 0.26 | 0.45 | 0.61 | 0.71 | 0.79 | 0.85 | 0.90 | 0.94 | 0.96 | 0.98 | 0.99 | 1.00 | 1.00 | 1.00 |
|  |  |  |  |  |  |  |  |  |  |  |  |  |  |  |
| Loads: |  |  |  |  |  |  |  |  |  |  |  |  |  |  |
|  | PC1 | PC2 | PC3 | PC4 | PC5 | PC6 | PC7 | PC8 | PC9 | PC10 | PC11 | PC12 | PC13 | PC14 |
| mean_itero | -0.33 | 0.24 | -0.55 | 0.43 | -0.12 | -0.31 | -0.25 | -0.34 | 0.07 | 0.22 | 0.05 | 0.00 | -0.01 | 0.00 |
| mean_curve | -0.65 | 0.24 | 0.23 | -0.31 | 0.13 | -0.50 | 0.11 | 0.23 | 0.07 | 0.11 | -0.08 | **-0.16** | 0.00 | -0.01 |
| mean_agemat | -0.58 | 0.46 | -0.46 | 0.09 | 0.14 | -0.09 | 0.26 | 0.28 | -0.07 | -0.03 | 0.21 | 0.12 | 0.00 | 0.01 |
| mean_matMLE | -0.82 | 0.12 | -0.04 | -0.15 | 0.38 | 0.23 | -0.01 | -0.04 | 0.10 | 0.10 | -0.20 | **0.16** | 0.00 | 0.01 |
| mean_reprate | -0.43 | -0.13 | 0.48 | -0.33 | **0.49** | 0.09 | -0.36 | -0.16 | -0.15 | 0.02 | 0.17 | -0.04 | -0.01 | 0.00 |
| mean_gentime | -0.64 | 0.20 | -0.45 | 0.24 | 0.03 | 0.40 | 0.19 | -0.10 | -0.22 | -0.04 | -0.05 | **-0.19** | -0.01 | 0.01 |
| mean_sexrep | -0.12 | -0.79 | -0.44 | -0.02 | 0.23 | 0.10 | 0.02 | 0.03 | 0.28 | -0.01 | 0.07 | -0.06 | 0.12 | 0.01 |
| mean_grow | -0.05 | -0.81 | -0.48 | -0.27 | 0.03 | 0.01 | 0.10 | 0.02 | 0.07 | 0.03 | 0.02 | -0.01 | -0.15 | -0.03 |
| mean_retrog | -0.24 | -0.67 | -0.40 | -0.20 | -0.18 | -0.28 | -0.19 | 0.07 | -0.35 | -0.04 | -0.09 | 0.06 | 0.05 | 0.01 |
| mean_juvsurv | -0.12 | 0.36 | -0.27 | -0.47 | **-0.48** | 0.34 | -0.36 | 0.26 | 0.07 | 0.12 | 0.03 | -0.03 | 0.00 | 0.01 |
| mean_adultsurv | -0.84 | 0.09 | 0.04 | 0.12 | -0.21 | -0.10 | -0.25 | -0.04 | 0.17 | -0.36 | -0.01 | 0.01 | -0.02 | -0.02 |
| mean_fec | 0.13 | -0.24 | 0.07 | 0.76 | 0.28 | 0.06 | -0.33 | 0.40 | -0.03 | 0.04 | -0.04 | -0.03 | -0.03 | -0.01 |
| mean_Eadultsurv | -0.61 | -0.41 | 0.48 | 0.21 | -0.33 | 0.15 | 0.15 | 0.00 | -0.04 | 0.12 | 0.04 | 0.05 | 0.04 | -0.08 |
| mean_Ejuvsurv | 0.56 | 0.47 | -0.54 | -0.20 | 0.32 | -0.01 | -0.11 | -0.01 | -0.03 | -0.07 | -0.04 | 0.00 | 0.04 | -0.09 |

Abbreviations: mean_itero = degree of iteroparity, mean_curve = type of survivorship curve, mean_agemat = age at maturity, mean_matMLE = mature life expectancy, mean_reprate = net reproductive rate, mean_gentime = generation time, mean_sexrep = mean sexual reproduction, mean_grow = progressive growth, mean_retrog = and retrogressive growth, mean_juvsurv = juvenile survival, mean_adultsurv = adult survival, mean_fec = average fecundity, mean_Eadultsurv = elasticity of adult survival, and mean_Ejuvsurv = elasticity of juvenile survival.

II. Trees

There were no important predictors from the crf, as the highest variable importance value did not exceed twice the magnitude of the largest negative value, which represents random variation.

| Importance |  |  |  |  |  |  |  |  |  |  |  |  |  |
| --- | --- | --- | --- | --- | --- | --- | --- | --- | --- | --- | --- | --- | --- |
|  | PC1 | PC2 | PC3 | PC4 | PC5 | PC6 | PC7 | PC8 | PC9 | PC10 | PC11 | PC12 | PC13 |
| Standard deviation | 1.68 | 1.54 | 1.35 | 1.13 | 1.06 | 0.89 | 0.86 | 0.81 | 0.71 | 0.60 | 0.53 | 0.44 | 0.27 |
| Proportion of Variance | 0.22 | 0.18 | 0.14 | 0.10 | 0.09 | 0.06 | 0.06 | 0.05 | 0.04 | 0.03 | 0.02 | 0.02 | 0.01 |
| Cumulative Proportion | 0.22 | 0.40 | 0.54 | 0.64 | 0.72 | 0.78 | 0.84 | 0.89 | 0.93 | 0.96 | 0.98 | 0.99 | 1.00 |
|  |  |  |  |  |  |  |  |  |  |  |  |  |  |
| Loads: |  |  |  |  |  |  |  |  |  |  |  |  |  |
|  | PC1 | PC2 | PC3 | PC4 | PC5 | PC6 | PC7 | PC8 | PC9 | PC10 | PC11 | PC12 | PC13 |
| mean_itero | -0.25 | 0.85 | -0.10 | 0.18 | -0.12 | 0.02 | -0.03 | -0.21 | -0.09 | 0.04 | 0.00 | 0.32 | 0.01 |
| mean_curve | -0.42 | -0.40 | -0.46 | -0.27 | 0.41 | 0.09 | -0.23 | 0.13 | 0.10 | -0.31 | -0.08 | 0.15 | 0.04 |
| mean_agemat | -0.18 | 0.25 | -0.44 | -0.58 | -0.15 | -0.05 | 0.30 | 0.47 | -0.09 | 0.18 | 0.04 | 0.04 | 0.01 |
| mean_matMLE | -0.45 | -0.47 | -0.36 | 0.05 | -0.33 | 0.24 | -0.30 | -0.10 | 0.28 | 0.28 | 0.14 | 0.03 | 0.00 |
| mean_reprate | -0.01 | -0.80 | -0.13 | 0.06 | -0.07 | -0.06 | 0.35 | -0.20 | -0.28 | -0.08 | 0.26 | 0.10 | 0.02 |
| mean_gentime | -0.42 | 0.60 | -0.47 | 0.05 | -0.04 | -0.12 | -0.25 | -0.05 | -0.17 | -0.16 | 0.25 | -0.19 | -0.01 |
| mean_sexrep | 0.73 | -0.02 | -0.58 | 0.13 | 0.24 | -0.10 | 0.02 | -0.02 | 0.08 | 0.04 | 0.03 | 0.05 | -0.20 |
| mean_grow | 0.70 | 0.05 | -0.54 | 0.06 | 0.14 | -0.35 | -0.01 | -0.15 | 0.11 | 0.11 | -0.04 | -0.02 | 0.17 |
| mean_retrog | 0.20 | 0.15 | -0.36 | 0.62 | -0.03 | 0.53 | 0.25 | 0.24 | 0.02 | -0.09 | -0.01 | -0.04 | 0.05 |
| mean_juvsurv | -0.61 | 0.10 | 0.18 | 0.35 | 0.14 | -0.39 | 0.30 | 0.15 | 0.41 | -0.05 | 0.12 | 0.02 | -0.01 |
| mean_adultsurv | -0.63 | 0.09 | -0.38 | -0.19 | 0.09 | 0.12 | 0.38 | -0.44 | 0.05 | 0.02 | -0.20 | -0.12 | -0.03 |
| mean_fec | 0.50 | 0.12 | -0.03 | -0.31 | -0.68 | 0.01 | 0.09 | -0.10 | 0.27 | -0.30 | 0.02 | 0.02 | -0.01 |
| mean_Eadultsurv | 0.34 | 0.37 | 0.30 | -0.41 | 0.49 | 0.35 | 0.10 | -0.15 | 0.19 | 0.04 | 0.25 | 0.00 | 0.03 |

III. Birds

The most important predictors from the crf were PCs 4 and 13. Based on loadings for PC4 and the partial dependence plot, there is a positive relationship so that higher values for sexual reproduction, progressive growth, and elasticity for adult survival were associated with higher-risk IUCN statuses. Based on loadings for PC13 and the partial dependence plot, there is a negative relationship so that higher values for iteroparity and lower generation time were associated with higher-risk IUCN statuses.

| Importance |  |  |  |  |  |  |  |  |  |  |  |  |  |
| --- | --- | --- | --- | --- | --- | --- | --- | --- | --- | --- | --- | --- | --- |
|  | PC1 | PC2 | PC3 | PC4 | PC5 | PC6 | PC7 | PC8 | PC9 | PC10 | PC11 | PC12 | PC13 |
| Standard deviation | 2.38 | 1.48 | 1.38 | 0.98 | 0.90 | 0.83 | 0.53 | 0.47 | 0.31 | 0.27 | 0.26 | 0.12 | 0.10 |
| Proportion of Variance | 0.44 | 0.17 | 0.15 | 0.07 | 0.06 | 0.05 | 0.02 | 0.02 | 0.01 | 0.01 | 0.01 | 0.00 | 0.00 |
| Cumulative Proportion | 0.44 | 0.61 | 0.75 | 0.83 | 0.89 | 0.94 | 0.96 | 0.98 | 0.99 | 0.99 | 1.00 | 1.00 | 1.00 |
|  |  |  |  |  |  |  |  |  |  |  |  |  |  |
| Loads: |  |  |  |  |  |  |  |  |  |  |  |  |  |
|  | PC1 | PC2 | PC3 | PC4 | PC5 | PC6 | PC7 | PC8 | PC9 | PC10 | PC11 | PC12 | PC13 |
| mean_itero | -0.60 | -0.58 | 0.38 | -0.02 | 0.14 | -0.27 | -0.22 | 0.09 | 0.01 | -0.07 | 0.08 | 0.02 | **-0.04** |
| mean_curve | -0.75 | 0.02 | -0.09 | -0.12 | -0.54 | -0.26 | 0.14 | 0.11 | -0.08 | 0.10 | -0.02 | 0.00 | -0.03 |
| mean_agemat | -0.78 | -0.31 | -0.10 | -0.11 | 0.29 | 0.30 | 0.09 | **0.24** | 0.10 | 0.04 | -0.11 | 0.00 | -0.01 |
| mean_matMLE | -0.74 | 0.33 | -0.51 | -0.24 | 0.03 | -0.06 | -0.03 | 0.03 | 0.04 | -0.08 | 0.08 | -0.08 | 0.00 |
| mean_reprate | -0.26 | 0.62 | -0.64 | -0.24 | 0.14 | -0.15 | -0.18 | -0.04 | 0.06 | 0.07 | 0.00 | 0.06 | -0.01 |
| mean_gentime | -0.78 | -0.55 | -0.13 | -0.16 | 0.13 | -0.05 | 0.05 | 0.00 | -0.09 | 0.06 | 0.08 | 0.02 | **0.07** |
| mean_sexrep | -0.77 | 0.36 | 0.07 | **0.39** | 0.13 | -0.06 | -0.23 | 0.04 | -0.14 | -0.01 | -0.11 | -0.02 | 0.01 |
| mean_grow | -0.59 | 0.32 | -0.02 | **0.59** | 0.26 | -0.23 | 0.28 | -0.01 | 0.05 | 0.00 | 0.06 | 0.01 | -0.01 |
| mean_retrog | -0.35 | -0.71 | -0.50 | 0.06 | 0.10 | -0.05 | 0.03 | **-0.31** | -0.01 | 0.01 | -0.07 | -0.02 | -0.03 |
| mean_juvsurv | -0.74 | 0.29 | 0.20 | -0.03 | 0.05 | 0.54 | 0.00 | -0.11 | -0.09 | 0.04 | 0.09 | 0.01 | -0.03 |
| mean_adultsurv | -0.87 | 0.22 | 0.22 | -0.24 | -0.19 | 0.02 | 0.12 | -0.10 | 0.02 | -0.18 | -0.07 | 0.04 | 0.01 |
| mean_fec | 0.75 | 0.01 | -0.52 | -0.13 | 0.27 | -0.02 | 0.14 | 0.13 | -0.18 | -0.09 | 0.00 | 0.02 | -0.02 |
| mean_Eadultsurv | -0.05 | -0.30 | -0.66 | **0.46** | -0.41 | 0.26 | -0.10 | 0.09 | 0.03 | -0.06 | 0.04 | 0.03 | 0.01 |

IV. Mammals

The most important predictor from the crf was PC1. Based on loadings for PC13 and the partial dependence plot, there is a negative relationship so that longer generation time, longer mature life expectancy, and later age at maturation were associated with higher-risk IUCN statuses.

| Importance |  |  |  |  |  |  |  |  |  |  |  |  |  |
| --- | --- | --- | --- | --- | --- | --- | --- | --- | --- | --- | --- | --- | --- |
|  | PC1 | PC2 | PC3 | PC4 | PC5 | PC6 | PC7 | PC8 | PC9 | PC10 | PC11 | PC12 | PC13 |
| Standard deviation | 1.93 | 1.81 | 1.31 | 1.18 | 1.05 | 0.85 | 0.53 | 0.47 | 0.46 | 0.42 | 0.33 | 0.24 | 0.12 |
| Proportion of Variance | 0.29 | 0.25 | 0.13 | 0.11 | 0.08 | 0.06 | 0.02 | 0.02 | 0.02 | 0.01 | 0.01 | 0.00 | 0.00 |
| Cumulative Proportion | 0.29 | 0.54 | 0.67 | 0.78 | 0.86 | 0.92 | 0.94 | 0.96 | 0.97 | 0.99 | 0.99 | 1.00 | 1.00 |
|  |  |  |  |  |  |  |  |  |  |  |  |  |  |
| Loads: |  |  |  |  |  |  |  |  |  |  |  |  |  |
|  | PC1 | PC2 | PC3 | PC4 | PC5 | PC6 | PC7 | PC8 | PC9 | PC10 | PC11 | PC12 | PC13 |
| mean_itero | 0.58 | 0.37 | -0.25 | 0.59 | 0.03 | -0.01 | 0.18 | 0.08 | 0.23 | -0.03 | 0.10 | -0.06 | 0.02 |
| mean_curve | -0.13 | -0.87 | -0.24 | 0.08 | 0.10 | -0.11 | 0.26 | -0.15 | 0.04 | 0.19 | 0.00 | 0.09 | 0.01 |
| mean_agemat | **0.77** | 0.18 | 0.22 | -0.13 | -0.09 | 0.47 | -0.06 | -0.24 | -0.03 | 0.08 | 0.10 | -0.01 | 0.03 |
| mean_matMLE | **0.74** | -0.52 | 0.25 | 0.13 | 0.14 | -0.02 | -0.11 | 0.21 | -0.03 | 0.08 | -0.11 | 0.01 | 0.07 |
| mean_reprate | 0.23 | -0.53 | 0.37 | -0.61 | 0.09 | -0.28 | -0.08 | 0.04 | 0.20 | 0.01 | 0.15 | -0.02 | -0.01 |
| mean_gentime | **0.83** | -0.41 | 0.13 | 0.12 | -0.03 | 0.29 | 0.01 | 0.10 | 0.04 | 0.02 | -0.05 | 0.04 | -0.09 |
| mean_sexrep | -0.50 | -0.18 | 0.70 | 0.28 | -0.23 | 0.04 | -0.03 | -0.15 | 0.23 | -0.05 | -0.13 | -0.02 | 0.00 |
| mean_grow | -0.40 | -0.33 | 0.63 | 0.34 | -0.37 | 0.02 | 0.12 | 0.13 | -0.18 | 0.01 | 0.15 | 0.01 | 0.00 |
| mean_retrog | -0.58 | 0.08 | -0.30 | -0.38 | -0.46 | 0.36 | 0.04 | 0.18 | 0.13 | 0.15 | -0.02 | -0.03 | 0.01 |
| mean_juvsurv | 0.58 | 0.47 | 0.21 | -0.45 | -0.28 | -0.08 | 0.27 | 0.02 | 0.00 | -0.15 | -0.06 | 0.09 | 0.02 |
| mean_adultsurv | 0.35 | -0.81 | -0.25 | -0.14 | -0.27 | -0.06 | 0.11 | -0.07 | -0.10 | -0.09 | -0.06 | -0.16 | 0.00 |
| mean_fec | -0.42 | 0.02 | 0.35 | -0.21 | 0.71 | 0.31 | 0.22 | 0.06 | -0.02 | -0.03 | -0.02 | -0.06 | 0.00 |
| mean_Eadultsurv | -0.29 | -0.78 | -0.35 | 0.05 | 0.04 | 0.31 | -0.08 | 0.02 | 0.05 | -0.26 | 0.06 | 0.08 | 0.02 |

Literature cited

1. Revell LJ. phytools: an R package for phylogenetic comparative biology (and other things). Methods Ecol Evol. 2012;3(2):217–23.

2. Strobl C, Boulesteix AL, Kneib T, Augustin T, Zeileis A. Conditional variable importance for random forests. BMC Bioinformatics. 2008;9:1–11.

3. Strobl C, Malley J, Tutz G. An Introduction to Recursive Partitioning: Rationale, Application, and Characteristics of Classification and Regression Trees, Bagging, and Random Forests. Psychol Methods. 2009;14(4):323–48.

4. Capdevila P, Beger M, Blomberg SP, Hereu B, Linares C, Salguero-Gómez R. Longevity, body dimension and reproductive mode drive differences in aquatic versus terrestrial life-history strategies. Funct Ecol. 2020;34:1613–1625.

5. Paniw, M., Ozgul, A. and Salguero-Gómez, R. Interactive life-history traits predict sensitivity of plants and animals to temporal autocorrelation. Ecol Lett. 2018;21:275–286.

6. Salguero-Gómez R, Jones OR, Jongejans E, Blomberg SP, Hodgson DJ, Mbeau-Ache C, et al. Fast-slow continuum and reproductive strategies structure plant life-history variation worldwide. Proc Natl Acad Sci. 2016;113(1):230–5.
